# Valorization of Grape Pomace as a Renewable Source of Techno-Functional and Antioxidant Pectins

**DOI:** 10.3390/antiox12040957

**Published:** 2023-04-19

**Authors:** Roberto Megías-Pérez, Alvaro Ferreira-Lazarte, Mar Villamiel

**Affiliations:** 1 Instituto de Investigación en Ciencias de la Alimentación (CIAL) (CSIC-UAM), Nicolás Cabrera, 9, Campus de la Universidad Autónoma de Madrid, 28049 Madrid, Spain; eljijo1981@hotmail.com (R.M.-P.); alvaro.ferreira@csic.es (A.F.-L.); 2 Singapore Institute of Food and Biotechnology Innovation, Agency for Science, Technology and Research, 31 Biopolis Way, #01-02, Nanos, Singapore 138669, Singapore

**Keywords:** grape pomace, pectin, conventional extraction, celluclast, antioxidant

## Abstract

The food industry’s increasing demand for new functional ingredients that meet both organoleptic and healthy requirements has driven the exploration of new sources of functional ingredients in agro-industrial by-products. The aim of this work was to valorize grape pomace (*Vitis vinifera* L. garnacha) as a source of pectins using food-grade extracting agents. Obtained pectins were evaluated for monomeric composition, methyl esterification, molecular weight, water retention, oil-holding capacity, and antioxidant properties. The relatively soft extraction conditions used permitted obtaining low methoxyl pectin (10–42%) enriched in homogalacturonan (38–45%) or rhamnogalacturonan (33–41%) with different branching degrees, molecular weight, and fewer impurities than those found in the scarce previous literature. The relationship between structure and functionality was studied. Among the different pectins obtained, the sample derived from the extraction with sodium citrate could resume the best characteristics, such as pectin purity and higher water retention and oil holding capacity. These results underscore the relevance of grape pomace as a viable alternative source of pectin.

## 1. Introduction

Pectins are plant cell wall polysaccharides with well-known applications as emulsifiers, stabilizers, and gelling and thickening agents. Recently, there has been an increased interest in pectin due to its biological properties, such as potential prebiotic, immunoregulatory, anti-inflammatory, antibacterial, antioxidant, and hypoglycemic properties [1].

Pectins have a complex structure consisting of different structural subunits, with the presence of a structural subunit called homogalacturonan (HG) as a common feature. HG consists of a polymer of galacturonic acid (GalA) units linked through α-1,4 glycosidic bonds, where the carboxyl group of GalA can be methyl-esterified in the hydroxyl group at the C-6 carboxyl and/or O-acetylated at O-2/O-3 positions. HG is linked to other subunits, such as rhamnogalacturonan I (RGI) and II (RGII). RGI consists of a backbone of the repeating disaccharide [-α-D-GalA-1,2-α-L-Rha-1-4-]n, with variable branching (side chains of arabinan, galactan, and arabinogalactans) attached at C-4 from the rhamnose residues. RGII has a complex structure consisting of 12 different types of sugars found in over 20 different linkages [2]. Moreover, it has been identified that the structure of pectin varies depending on extraction conditions and plant tissues, adding an additional level of complexity [3].

Industrially, pectins are mainly extracted from agri-food by-products (mainly citrus peel) using methods involving strong acids and high temperatures for a long time. However, these methods have significant drawbacks, such as elevated manufacturing costs, excess residues, and pectin structure degradation [4]. Currently, environmentally friendly alternatives, such as sodium citrate [5] or deep eutectic solvents (DES) [6], which can preserve pectin structure, have been proposed as milder extraction conditions and food-grade extraction agents. In addition, the development of other techniques, such as enzyme-assisted extraction (EAE), has brought new opportunities to valorize agri-food by-products as a source of pectin by developing new processes with greater extraction yields [7].

Grape pomace (GP) constitutes the most abundant waste in the wine industry, which comprises skin, seeds, and other solid parts, with more than 20% of pectic substances [8]. Despite the potential of GP as an unconventional source of pectin, only a few studies have been published on the extraction of pectin from different red and white grape varieties, with dissimilarities in the results of yields and structural characterization [9,10,11,12]. Furthermore, no evaluation of the properties of pectin has been developed.

The limited studies on pectins obtained from GP have employed methodologies that may yield pectins with an altered structure, as in the case of pectins obtained by ultrasound [9] or with strong solvents such as boiling HNO_3_ [10] and tartaric acid/ammonium oxalate [11]. No comparative investigation has been carried out to explore how the structural and/or functional characteristics could vary depending on the type of solvent used in the extraction.

Therefore, this present study aims to explore the potential of GP as a source of pectin using various extraction solvents, such as oxalic, citric, malic, and nitric acids, sodium citrate, choline chloride, and a commercial enzymatic preparation of cellulase. It is hypothesized that this approach will yield different types of pectins, with distinct functional properties, based on the extraction conditions. This study will, therefore, involve a preliminary screening to identify the optimal extraction conditions for specific functional properties. To this end, an in-depth chemical, physicochemical, and functional characterization of GP pectin will be carried out.

## 2. Material and Methods

### 2.1. Material and Samples

Xylose (Xyl), arabinose (Ara), rhamnose (Rha), galactose (Gal), glucose (Glc), mannose (Man), galacturonic acid (GalA), pullulan standards, citric acid (CA), nitric acid (NA), oxalic acid (OA), malic acid (MA), sodium citrate (SC), choline chloride, standard β-phenyl-glucoside, and NH_4_Ac were acquired from Sigma-Aldrich (St. Louis, MO, USA). Ethanol 96% was purchased from Alcoholes Montplet (Barcelona, Spain). Celluclast^®^1.5 L was donated by Novozymes (Bagsvaerd, Denmark).

The by-product grape (*Vitis Vinifera* L., garnacha) pomace (peels and seeds) was a gift from the winery “A Pie de Tierra” from Méntrida, Toledo (Spain) (season 2020).

### 2.2. Pectin Obtainment and Characterisation

**Figure 1:**
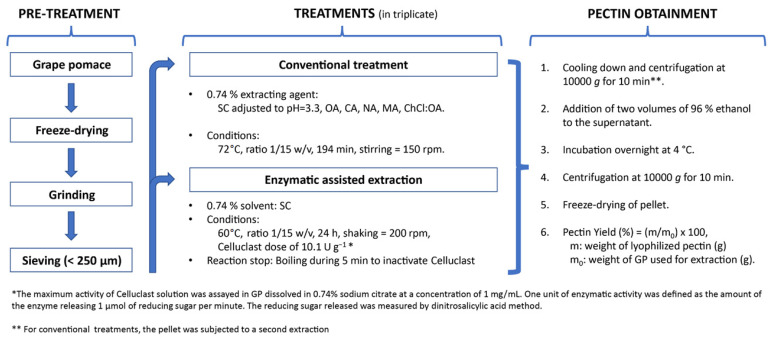
Overview of the different treatments and conditions. SC, sodium citrate; OA, oxalic acid; CA, citric acid; NA, nitric acid; MA, malic acid.

The different treatments and conditions performed in this study are summarized in Figure 1. The DES ChCl/OA was synthesized according to the method described previously [13]. For each extracting agent, two consecutive treatments were implemented.

Aqueous solutions (10 mg/mL) of GP or pectin were used to determine the pH with a pH meter (Mettler Toledo GmBH, Schwerzenbach, Switzerland).

Water activity (a_w_) was measured with A_W_ Sprint TH-500 instrument (Novasina, Pfäffikon, Switzerland).

The dry matter (DM) was determined gravimetrically in an oven at 102 °C until constant weight.

### 2.3. Structural Characterisation

#### 2.3.1. Estimation of Molecular Weight Distribution

Pectin solutions in water (1 mg/mL) were analyzed by HPSEC-ELSD on an Agilent Technologies 1220 Infinity LC chromatograph coupled with a 1260 Infinity ELSD detector (Agilent Technologies, Boeblingen, Germany). Chromatographic separation was achieved with 2 TSK-Gel columns G5000 PWXL (7.8 mm × 300 mm, 10 μm) and G2500 PWXL (7.8 mm × 300 mm, 6 μm) connected in series. Elution of the samples was performed under isocratic conditions of 0.01 M NH_4_Ac for 50 min at 30 °C. Injection volume was set to 50 µL. The flow rate used was 0.5 mL/min.

The estimation of average Mw was calculated by the external calibration method using solutions of commercial pullulan standards (M_w_ 0.342–805 kDa) [14].

#### 2.3.2. Monosaccharide Composition

Pectins were hydrolyzed with trifluoroacetic acid (TFA) 2M at 110 °C at a concentration of 20 mg/mL for 4 h. After that, the samples were derivatized by treating with hydroxylamine chloride in pyridine (2.5 %, *w*/*v*), hexamethyldisilazane and TFA.

Samples were analyzed by GC-FID (7890A gas chromatograph; Agilent Technologies, Wilmington, DE, USA) using a DB-5HT capillary column (30 m × 0.32 mm × 0.10 μm) (J&W Scientific, Folson, CA, USA). Oven temperature program was increased from 150 °C to 165 °C at 1 °C/min, then increased at a rate of 10 °C/min to 200 °C and up to 380 °C at a heating rate of 50 °C/min. Injector temperature was 280 °C, and detector temperature was 350 °C; oven temperature was increasing from 150 °C to 165 °C at 1 °C/min and up to 300 °C at a heating rate of 10 °C/min.

Quantitation was performed by the internal standard method, the standard being β-phenyl-glucoside (0.05% *w*/*v*). Response factors were calculated after analysis of standard solutions (glucose, mannose, rhamnose, arabinose, galactose, GalA, and xylose) in the expected concentration range of the samples (0.01–1 mg/mL) [15].

The *HG* content was determined using the following equation:

HG (%)=GalA−Rha

The *RGI* backbone was characterized based on several parameters such as *RGI* content, degree of branching (*DB*), and extent of branching (*EB*) [16]:

RGI content (%)=2∗Rha+Ara+Gal

$$DB=\frac{GalA}{Rha}$$

$$EB=\frac{Ara+Gal}{Rha}$$

Furthermore, the linearity of the pectin backbone (*LP*) was calculated as follows:

(1) $$LP=\frac{GalA}{Rha+Ara+Gal}$$

Pectin purity (*PP*) was determined using the formula:

(2) $$PP=\frac{GalA+Rha+Ara+Gal}{Glu+Man}$$

#### 2.3.3. Degree of Methyl Esterification (DM)

Pectins were analyzed using Attenuated Total Reflectance Fourier Transform Infrared Spectroscopy (ATR-FTIR) (Perkin Elmer, Madrid, Spain). Data were collected in absorbance mode using a frequency range of 4000–450 cm^−1^ and resolution of 4 cm^−1^ (mid infrared region) with 20 co-added scans. The DM was determined as the average ratio of the peak area at 1739 cm^−1^ (COO–R) over the sum of the peak areas of 1739 cm^−1^ (COO–R) and 1637 cm^−1^ (COO−).

### 2.4. Techno-Functional and Bioactive Properties

Water retention capacity (WRC): Pectins were incubated with deionized water (1:10 *w*/*v*) for 24 h with continuous agitation. Then, samples were centrifuged at 1006× *g* (Eppendorf, 5804R, Eppendorf Ibérica, Madrid, Spain) for 30 min. WRC was expressed as mL of water held by 1 g of pectin.

Oil holding capacity (OHC): Pectins were dissolved with corn oil (1:20 *w*/*v*) and incubated for 30 min with continuous agitation. Then, samples were centrifuged (Eppendorf, 5804R) at 1006× *g* for 30 min. After centrifugation, the volume of the supernatant was measured. The OHC was expressed as g of oil retained for g of pectin.

Total phenolic content: Aqueous pectin solutions (5 mg/mL) were used to determine the total phenolic content (TPC) by the Folin–Ciocalteu method [17].

Antioxidant capacity: The antioxidant capacity of each pectin was determined as the IC50 value. From each pectin, different aqueous solutions (0.5–10 mg/mL) were analyzed following the DPPH (2,2-Diphenyl-1-picrylhydrazyl) method described by Calvete-Torre et al. [15]. The relationship between the percentage of inhibition and sample concentration (mg/mL) was plotted and subsequently used to determine the IC50 value of each sample.

### 2.5. Statistical Analysis

Each assay was performed in triplicate. Statistical analyses were conducted using IBM SPSS for Windows (version 17.0, SPSS Inc., Armonk, NY, USA), and *t*-Student test was used for statistical pairwise comparison.

## 3. Results and Discussion

### 3.1. Yields and Physicochemical Characterisation of Pectin

Considering two consecutive extractions from the grape pomace (GP), the yield values obtained were 4.53, 4.4, 4.21, 3.61, 3.45, 2.76 and 2.8% for DES ChCl/OA, citric acid (CA), oxalic acid (OA), sodium citrate (SC), malic acid (MA), nitric acid (NA), and enzymatic treatments (EAE), respectively.

Yields obtained after citric acid extraction showed different values compared to those observed in previous works for GP. Minjares-Fuentes et al. [9] obtained yields of 32.3% (g obtained per 100 g of GP, fresh weight) after extraction with the same extractant solution at pH 2.0, 75 °C, and 60 min. Spinei and Oroian [12,18] and Xu et al. [19] reported yields of 9–11% pectin from different grape pomace varieties after microwave-assisted or ultrasound-assisted extraction, with a citric acid solution at different pH values, after 2 or 3 h of treatment. However, the moisture of most of these pectins was not analyzed, and the duration of the drying, 12 h at room temperature, could suggest the possibility of high moisture remaining in the pectin. Colodel et al. [10] obtained yield values of 11% pectin after extraction, using the alcohol-insoluble residue as starting material instead of raw GP, with boiling nitric acid at pH 2.0 for 135 min. However, most studies on the extraction of pectin from GP have reported low yields in accordance with the obtained in this study [8]. Vasquez et al. [20] evaluated the effect of different drying methods for grape pomace material on the acidic extraction of pectin using HCl as solvent (pH 1.5, 50 °C), obtaining yields of 2.5–3.1% of freeze-dried pectin as obtained in our study. Moreover, Sousa et al. [21] analyzed the composition of grape pomace of the *Benitaka* variety and found 3.92% pectin. Similar values were also obtained by Deng et al. [22] for 5 different varieties ranging from 3.2 to 5.6%. Ferreira et al. [23] reported yields of 1–2% grape pomace pectin after aqueous KOH extraction and purification by ultrafiltration and microfiltration. In general, yields of extracted pectin increased when pH decreased to 1.0 and at high temperatures (90 °C), which can be related to the breakage of hydrogen bonds and ester interconnections between the cell wall and pectin at these conditions, promoting the release of these polysaccharides [8,11,18].

Table 1 shows pH, a_w_ and protein content values. pH influences the binding capacity of pectin to divalent cations, affecting technological properties such as gelation [24]. At pH values of at least one log unit above, the pectin pKa (2.8–4.1) is required to ensure more than 50% dissociated carboxyl groups and, therefore, a sufficient charge density for the formation of ionic cross-links. A pH of 5 can be optimal for the binding capacity with Ca^2+^ and Zn^2+^, whereas a pH higher than 6 might decrease the binding capacity for these cations [25,26]. Pectin obtained after acidic extraction (Table 1) showed lower pH values (2.6–4.4) compared to samples obtained by enzymes (6.6). In general, the results indicate the anionic character of the pectin, suggesting the suitability of most samples (obtained with CA, MA, ChCl/OA, and SC) for their use in gelation processes [27].

A_w_ is an indicator used to evaluate the moisture balance produced by the free water available in a food product [28]. Lower a_w_ values indicate a lower probability of microbiological growth and lead to the longer shelf life of the food product. As shown in Table 1, obtained pectin had low a_w_ values (0.173–0.405), which guarantees their suitability as food additives. These values were similar to those obtained for pectin from berries by previous works [29].

Protein content plays a role in the emulsifying activity of pectins due to the hydrophobic character of the acyl and feruloyl ester groups that facilitate the formation and retention of fine droplets during emulsification [30]. As indicated in Table 1, the protein content of all studied samples was in the range of 1.3–5.4%, showing the highest value in the conventional treated (CT) samples obtained with OA.

### 3.2. Structural Characterisation

#### 3.2.1. Molecular Weight Distribution

**Figure 2:**
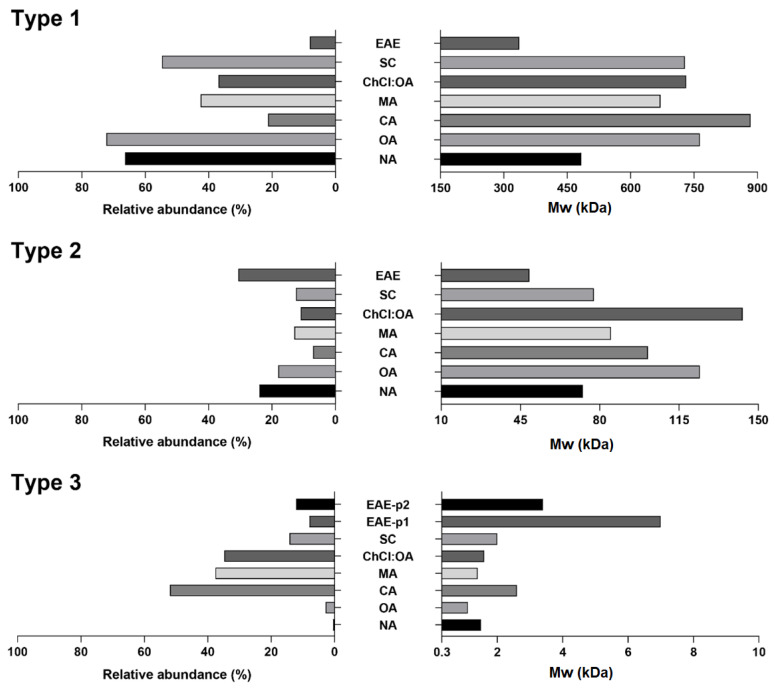
Average Mw values (kDa) and relative abundances (%) of the main type of fragments detected in the pectin samples under study. NA (Nitric Acid), OA (Oxalic Acid), CA (Citric Acid), MA (Malic Acid), ChCl/OA (DE Choline Chloride/Oxalic Acid), SC (Sodium Citrate), EAE (Enzymatic-Assisted Extraction), p1 (peak 1), p2 (peak 2).

Figure S1 shows an HPSEC-ELSD profile of the studied pectins. Although up to five peaks were detected, the chromatograms were divided into four sections: the most interesting for the abundance and the order of elution for types 1 and 2, respectively. Figure 2 shows the abundance and distribution for each extracting agent. Average Mw values of type 1 fragments from conventionally treated samples (483.1–883.6 kDa) were higher than those observed for EAE samples (336.3 kDa), which could be attributed to the different mechanisms involved in EAE, as observed in previous reports [31]. These values were also higher compared to those reported previously for extraction of pectin from GP employing boiling nitric acid (154.1 kDa) [10], hydrochloric acid (49–63 kDa) [20], tartaric acid or ammonium oxalate (4 peaks from 3.6 to 55.0 kDa) [11], UAE with citric acid (45.3–203.3 kDa) [9,18], or microwaves with citric acid (41–46 kDa) [12].

These differences could be ascribed to the moderate extracting conditions used in this study (72 °C, 194 min, and 0.74% extracting agent). Extracting conditions and the use of ultrasound or microwave assistance can affect the structure of pectins and, thereby, decrease their molecular weight [12]. Contrarily, Xu et al. [19] obtained molecular weights of 1553.27 and 865.85 kDa for pectin obtained from unfermented and fermented GP, respectively, using citric acid as extracting solvent, but these results could be misinterpreted since it has been pointed out that citric acid can be associated with pectin [32].

The Mw is correlated with its gel-forming, thickening, and stabilizing properties, which influence the utilization of pectin in the food industry. Values determined for conventionally treated samples could suggest that GP consists of long polymeric chains with potential good rheological properties to be used as a thickener or gelling agent [33].

Regarding type 2 fragments, all samples showed similar Mw values to those observed in previous reports [10,11,12,20] (Figure 2). For type 3 fragments, conventionally treated samples presented 1 peak corresponding to Mw values (1.1–2.6 kDa), whereas EAE samples had 2 peaks (3.4 kDa and 7 kDa). Sabater et al. [34] extracted pectin from artichoke by-products using the same enzyme preparation at 50 °C and found 3 peaks of 660, 105, and 4.8 kDa and were the second and the third the most abundant, indicating the suitability of Celluclast to produce oligosaccharides from pectin. Regarding relative abundance (Figure 2), conventionally treated samples showed a wide dispersion (0.5–51.9%), while EAE samples showed a narrower range (7.9–12.1%).

As an overview regarding Mw of the conventional samples, a certain degree of degradation can be inferred in pectin obtained with CA, MA, and ChCl/OA solvents based on the lower relative abundance of type 1 fragments (<50%) and the higher relative abundance of type 3 fragments (>30%).

#### 3.2.2. Monomeric Composition

Table 2 shows the monomeric composition of the different pectins under study. In general, the values determined for each monosaccharide were in the same order of magnitude as previous studies on GP [10,12,18,19] or grape skin [35].

GalA was found to be the most abundant monosaccharide in pectins (42.3–52.5%), highlighting its significance. The highest GalA content was observed with NA, while the lowest was obtained with the EAE. Determination of rhamnose, arabinose, and galactose confirmed the presence of RGI in the pectin structure. Remarkably, the arabinose values obtained with NA were significantly lower (*p* < 0.05) as compared to other samples, which might be attributed to the strong conditions produced by the acid [10].

In addition, xylose, glucose, and mannose monosaccharides were also quantified. The identification of xylose (1.8–4.5%) in all samples could suggest the presence of xylogalacturonan as a branch of the RGI subunit [36] or the presence of xyloglucan [37].

Moreover, glucose (12.0–20.8%) and mannose (1.6–3.4%) content, in the present and previous studies, could suggest the presence of other polymers, such as cellulose and hemicellulose and mannans, respectively. Although, the presence of starch should not be ruled out [10].

Table 3 shows the structural parameters inferred from the monosaccharide composition (formula Section 2.3.2.). HG was the main component in pectins obtained using NA, CA, OA and MA. In contrast, RGI was predominant in EAE samples as well as in those obtained using SC and ChCl/OA. In general, the data support the presence of arabinogalactan, galactan and/or arabinan branches attached to the RGI subunit. DB showed values of 7.2–10.2, and considerable variability in EB values was observed (3.0–6.9).

Pectins obtained using NA showed the highest value for LP backbone compared to the other pectins. These data could suggest that the strong conditions of NA could dramatically affect the structure of pectin, losing part of it as RGI. This is also supported by the lower content of RGI and the low value determined for the EB compared to the other pectins.

The purity of the pectins (PP) indicates the relationship between pectin and other co-extracted polymers. Samples showed 3.3–5.7 times more content of pectin than other non-pectic polysaccharides, with pectin extracted with SC being the purest. Calculated PP for pectin obtained by Colodel et al. [10], after treatment with boiling nitric acid, 135 min, pH 2, was 2.49, indicating the lower number of impurities in pectin obtained with the same acid in this study. Similarly, the PP of extracted pectin by oxalic acid, tartaric acid, and citric acid in previous reports showed values of 1.46, 1.25, and 4.12, respectively, showing the high purity of the obtained pectin [11,19].

### 3.3. Pectin Characterisation by ATR-FTIR

The ATR-FTIR spectra confirmed the presence of the characteristic functional groups in the pectin structure. Figures S2 and S3 show representative examples of the ATR-FTIR spectra. The bands located at 830 cm^−1^ and 922 cm^−1^ were assigned to the vibrations of the pyranose ring of the monosaccharide’s constituent of pectins. The band centered at 1018 cm^−1^, 1098 cm^−1^, and 1146 cm^−1^ were assigned to glycosidic linkages (C–O), (C–C), and (O–C–O), respectively, characteristics of pectins backbone vibrations [38].

The band located at 1440 cm-^1^ is assigned to the asymmetric stretching vibration modes of methyl esters, indicating the presence of methyl, methylene, and methoxyl groups on the structure [39].

Areas of the bands centered at 1739 cm^−1^ (stretching mode of the methyl esterified carbonyl groups) and 1631 cm^−1^ (antisymmetric stretching modes of the free COO carboxylic groups) were used to estimate DM values. Table 3 shows the estimated DM values of the different pectins under study. Among the CT samples, all DM values were low (10–42%) except for the pectins obtained with NA (92%). The lowest DM value (1%) was found in pectins obtained by EAE, probably due to the presence of pectin methyl esterase activity of Celluclast. These results underline the influence of solvent and type of extraction on the structure of pectins. Other authors have shown DM values in the range of 20.1–62.1% [9] and 43.3% [10], in line with the values here reported.

The presence of arabinan and galactans linked to pectin structure is supported by the bands centered at 1044 cm^−1^ and 1075 cm^−1^ [40], confirming the data suggested by the monosaccharide composition.

### 3.4. Techno-Functional and Antioxidant Properties

The application of pectins in the food industry is an interesting approach that can provide dual functionalities such as antioxidants and thickeners/stabilizers [41]. The structural characterization of grape pomace pectins was completed by the evaluation of techno-functional and antioxidant properties (Table 4).

WRC and OHC provide information on the ability of pectin to retain water or hold oil, respectively, useful abilities for the food industry to confer firmness and viscosity [42].

Regarding WRC, pectins showed values in the range of 2.4–21.3 mL/g, with samples obtained with SC and NA presenting the highest and lowest WRC values, respectively; these values were significantly different from the rest of the samples (*p* < 0.05). Based on the structural differences observed (NA, elevated linearity, and proportion of HG, whereas SC, more branched and with a predominance of RGI), the variability in WRC could be attributed to intrinsic factors like the hydrophobic character of samples [43].

Pectins showed OHC values in the range of 14.1–25.4 g/g; interestingly, samples with higher OHC values (EAE and CT NA samples) had lower Mw values for type 1 fragments. The differences observed could be attributed to the affinity of the pectins for the oil, which depends essentially on the chemical composition, specifically on the hydrophilic nature and the overall charge density of the polysaccharide [43].

WRC and OHC values were equal to or slightly higher than those values determined for pectin from berries (WRC = 0.3–11.8 mL/g and OHC = 5–17.8 g/g) [29]. Almost no data can be found on the functionality of GP pectin; only Spinei and Oroian [44], who carried out conventional, MAE, and pulsed UAE of pectin from GP, evaluated WRC and OHC, obtaining values of 5.39–14.35 mL/g and 2.19–3.24 g/g, respectively, highlighting the potential use of the pectin obtained in this study, given their high WRC and OHC.

On the other hand, pectins show the ability to reduce free radicals due to the presence of polyphenols in their structure and the effect of the pectin structure itself [41]. Polyphenols can be co-extracted with pectins, remaining bound to the structure. TPC determination in the pectins showed values in the range of 10.1–23.9 mg GAE/g pectin, similar to those (2.8–3.6%) of Colodel et al. [10]. The lowest values can be attributed to the strong extraction conditions. In this sense, the lowest TPC values corresponded to the samples obtained by NA (strong acid) or EAE (final step of 5 min boiling to enzyme inactivation).

In cucumber tree (Averrhoa bilimbi) pectin, although the TPC increased the antioxidant activity (DPPH, FRAP methods), the presence of hydroxyl groups and GalA scarcely methylated, which could contribute in a positive way to this property [45].

Regarding antioxidant activity, the IC50 is a quantitative measure indicating the concentration of pectin required to scavenge DPPH radicals by 50%. Lower IC50 values indicate higher antioxidant activity and vice versa. All assayed pectin presented antioxidant activity with IC50 values in the range of 3.1–6.9 mg/mL. The lowest IC50 values corresponded to ChCl/OA samples (3.1 mg/mL). In contrast, NA samples (lowest content of TPC and M_w_) showed significantly higher IC50 values than the other samples (*p* < 0.05), suggesting that structural changes cause the pectin to lose some of its antioxidant properties. The increase in M_w_ and –OH and –COOH groups could improve the antioxidant activity in DPPH assays [41]. It was previously reported that a higher content of uronic acids, lower neutral sugar content and a higher level of branching could lead to a higher antioxidant activity [46], as observed in previous reports [19,20,23].

## 4. Conclusions

The relatively soft extraction conditions used in this study permitted obtaining low methoxyl pectin enriched in homogalacturonan or rhamnogalacturonan regions. Fractions of low Mw were observed; however, in general, pectin samples showed a high Mw distribution (700 KDa average), indicating the suitability of these pectins as thickeners. The most abundant domain was HG in pectins obtained with nitric, citric, oxalic, and malic acids, whereas the other extracting agents gave rise to pectins with more proportion of RGI than HG. However, regardless of the extraction, it can be inferred that RGI was branched to arabinans, galactans, arabinogalactans, and xylogalactans, with different variability of branching. In addition, most of the samples were low methoxyl esterified, which makes these pectins potentially applicable as gelling agents without the addition of sucrose.

It can be concluded from the results that a wide range of structures with different functionalities can be obtained from grape pomace. Thus, pectins obtained with strong solvents such as nitric acid or by Celluclast could have a lower potential for application in the food industry. In contrast, high molecular weight pectins, such as those obtained with solvents like choline chloride/oxalic acid, oxalic acid and sodium citrate, seem to have more applications. Pectin obtained by sodium citrate could be highlighted not only for its good functional properties but also for its better level of purity. Therefore, the obtained results underline the appropriateness of grape pomace as an alternative source of pectin with techno-functional and/or bioactive properties that can be used as a food ingredient in a wide range of applications.

## Figures and Tables

**Table 1:** Physicochemical characterization of grape pomace (GP) pectins extracted using different methodologies. NA (Nitric Acid), OA (Oxalic Acid), CA (Citric Acid), MA (Malic Acid), ChCl/OA (DES Choline Chloride/Oxalic Acid), SC (Sodium Citrate), and EAE (enzymatic assisted extraction).

Treatment	Protein (%)	pH	a_w_
NA	2.7 ± 0.3 ^c^	2.6 ± 0.0 ^a^	0.275 ± 0.013 ^b^
OA	5.4 ± 0.5 ^d^	3.2 ± 0.0 ^b^	0.259 ± 0.029 ^b^
CA	2.2 ± 0.1 ^b^	3.7 ± 0.0 ^c^	0.405 ± 0.060 ^c^
MA	3.1 ± 0.3 ^c^	3.8 ± 0.1 ^c^	0.335 ± 0.050 ^b,c^
ChCl/OA	3.2 ± 0.2 ^c^	3.7 ± 0.1 ^c^	0.371 ± 0.023 ^c^
SC	1.6 ± 0.2 ^a^	4.4 ± 0.1 ^d^	0.173 ± 0.018 ^a^
EAE	1.3 ± 0.1 ^a^	6.6 ± 0.1 ^e^	0.260 ± 0.013 ^b^

Different letters within the same column indicate significant differences.

**Table 2:** Monomeric composition (%) of pectin extracted from grape pomace by (A) NA (Nitric Acid), OA (Oxalic Acid), CA (Citric Acid), MA (Malic Acid), ChCl/OA (DES Choline Chloride/Oxalic Acid), SC (Sodium Citrate), EAE (Enzymatic-Assisted Extraction).

Treatment	Xyl	Ara	Rha	Gal	Man	Glc	GalA
NA	3.6 ± 0.4^c,d^	3.2 ± 0.1 ^a^	5.3 ± 0.1 ^b^	12.8 ± 0.5 ^b^	1.7 ± 0.0 ^a^	20.8 ± 3.0 ^c^	52.5 ± 3.0 ^b,c^
OA	3.1 ± 0.2 ^c^	11.1 ± 1.0 ^b^	5.5 ± 0.4 ^b,c^	10.8 ± 1.4 ^a^	1.6 ± 0.1 ^a^	18.3 ± 0.9 ^b,c^	49.5 ± 2.8 ^b^
CA	3.0 ± 0.2 ^c^	14.7 ± 0.4 ^c^	4.6 ± 0.2 ^a^	11.5 ± 1.0 ^a,b^	2.4 ± 0.3 ^b^	16.2 ± 1.3 ^b^	47.4 ± 1.6 ^b^
MA	3.0 ± 0.4 ^c^	16.0 ± 1.1 ^c,d^	5.0 ± 0.5 ^a,b^	10.8 ± 1.3 ^a^	2.0 ± 0.3 ^a,b^	15.4 ± 0.7 ^b^	47.8 ± 1.4 ^b^
SC	4.4 ± 0.6 ^d^	18.4 ± 2.3 ^d^	5.9 ± 0.3 ^c^	10.9 ± 1.0 ^a^	2.2 ± 0.2 ^b^	12.0 ± 1.6 ^a^	46.2 ± 2.6 ^a,b^
ChCl/OA	2.3 ± 0.2 ^b^	16.4 ± 0.8 ^d^	4.3 ± 0.2 ^a^	13.9 ± 0.9 ^b,c^	3.4 ± 0.5 ^c^	16.4 ± 1.0 ^b^	43.2 ± 1.1 ^a^
EAE	1.8 ± 0.2 ^a^	16.7 ± 0.8 ^d^	5.9 ± 0.2 ^c^	12.6 ± 0.5 ^a,b^	7.1 ± 0.4 ^d^	13.7 ± 0.3 ^a^	42.3 ± 1.3 ^a^

Different letters within the same column indicate significant differences between sample groups (extracting method).

**Table 3:** Structural parameters inferred from the monomeric composition of pectin from grape pomace treated by (A) NA (Nitric Acid), OA (Oxalic Acid), CA (Citric Acid), MA (Malic Acid), ChCl/OA (DES Choline Chloride/Oxalic Acid), SC (Sodium Citrate), EAE (Enzymatic-Assisted Extraction). HG; Homogalacturonan, RGI; Rhamnogalacturonan-I, DB; Degree of Branching, LP; Linearity Pectin, PP; Pectin Purity, DM; Degree of Methyl esterification.

Treatment	HG (%)	RGI (%)	DB RGI	EB RGI	LP	PP	DM (%)
NA	47.2	26.6	9.8	3.0	34.6	3.3	92
OA	44.0	33.0	8.9	3.9	20.9	3.9	42
CA	42.8	35.5	10.2	5.6	19.3	4.2	10
MA	42.8	36.8	9.6	5.4	18.8	4.6	14
SC	40.3	41.1	7.8	5.0	19.3	5.7	24
ChCl/OA	38.9	39.0	10.0	6.9	20.9	3.9	27
EAE	36.4	41.1	7.2	5.0	22.0	3.7	1

**Table 4:** Functional properties of GP pectin. NA (Nitric Acid), OA (Oxalic Acid), CA (Citric Acid), MA (Malic Acid), ChCl/OA (DES Choline Chloride/Oxalic Acid), SC (Sodium Citrate), EAE (Enzymatic-Assisted Extraction). WRC; Water Retention Capacity, OHC; Oil-Holding Capacity, IC50; Half inhibitory concentration, TPC; Total Phenolic Content.

Treatment Type	WRC (mL/g)	OHC (g/g)	IC50 (mg/mL)	TPC (mg GAE/g Pectin)
NA	2.4 ± 0.3 ^a^	24.2 ± 1.4 ^c^	6.9 ± 0.7 ^d^	10.1 ± 1.3 ^a^
CA	11.9 ± 1.7 ^b^	14.1 ± 2.0 ^a^	3.8 ± 0.3 ^b^	21.7 ± 0.3 ^d^
MA	15.7 ± 1.5 ^c^	19.7 ± 2.4 ^b^	4.4 ± 0.2 ^c^	21.8 ± 2.4 ^d,e^
OA	12.4 ± 1.2 ^b^	18.8 ± 0.6 ^b^	3.7 ± 0.4 ^b^	23.3 ± 0.7 ^e^
SC	21.3 ± 2.4 ^d^	20.6 ± 1.3 ^b^	3.8 ± 0.2 ^b^	17.7 ± 1.7 ^c^
ChCl/OA	14.4 ± 0.5 ^c^	20.3 ± 1.1 ^b^	3.1 ± 0.1 ^a^	23.9 ± 0.9 ^e^
EAE	10.7 ± 1.0 ^b^	25.4 ± 2.0 ^c^	4.7 ± 0.5 ^c^	11.8 ± 0.2 ^b^

Different letters within the same column indicate significant differences between groups (extracting method).

## Data Availability

The supporting information can be downloaded from Supplementary Materials.

## Supplementary Materials

The following supporting information can be downloaded at: https://www.mdpi.com/article/10.3390/antiox12040957/s1. Figure S1. HPLC-ELSD chromatogram of GP pectin extracted with Oxalic Acid. In the figure are indicated the different types of fragments observed. Figure S2. ATR-FTIR spectra (signal between 4000–1500 cm^−1^) of pectin obtained with (A) Nitric Acid and (B) Oxalic Acid. Figure S3. ATR-FTIR spectra (signal between 1500–450 cm^−1^) of pectin obtained with (A) Nitric Acid and (B) Oxalic Acid.

## Abbreviations

GP	Grape Pomace
CT	Conventional Treatments
NA	Nitric Acid
MA	Malic Acid
OA	Oxalic Acid
CA	Citric Acid
SC	Sodium Citrate
ChCl	Choline Chloride
GalA	Galacturonic Acid
Mw	Molecular weight
DM	Degree of Methyl esterification
OHC	Oil-Holding Capacity
WRC	Water Retention Capacity
DB	Degree of Branching
EB	Extent of Branching
LP	Linearity Pectin
N-PP	Non-Pectic Polysaccharides
GalA	Galacturonic Acid
Rha	Rhamnose
Xyl	Xylose
Ara	Arabinose
Gal	Galactose
Glc	Glucose
Man	Mannose
HPSEC-ELSD	High-Performance Size-Exclusion Chromatography (HPSEC) coupled to Evaporative Light Scattering Detector (ELSD)
