# Supplementary material for: Valorization of Grape Pomace as a Renewable Source of Techno-Functional and Antioxidant Pectins
Source: Antioxidants (Basel). 2023 Apr 19;12(4):957. doi: 10.3390/antiox12040957 (PMC10136187; doi:10.3390/antiox12040957)
Supplement: Supplementary file 1 [file antioxidants-12-00957-s001.zip › antioxidants-2316454-supplementary.pdf]

Supplementary information of the manuscript

"Valorization of Grape Pomace as a Renewable Source of Techno-Functional and Antioxidant Pectins"

Roberto Megías-Pérez [1]

Alvaro Ferreira-Lazarte [1,2]

Mar Villamiel [1,*]

[1] Instituto de Investigación en Ciencias de la Alimentación (CIAL) (CSIC-UAM), Nicolás Cabrera, 9, Campus de la Universidad Autónoma de Madrid, 28049 Madrid, Spain

[2] Singapore Institute of Food and Biotechnology Innovation, Agency for Science, Technology and Research, 31 Biopolis Way, #01-02, Nanos, Singapore 138669, Singapore

* Correspondence: m.villamiel@csic.es; Tel.: 910017902

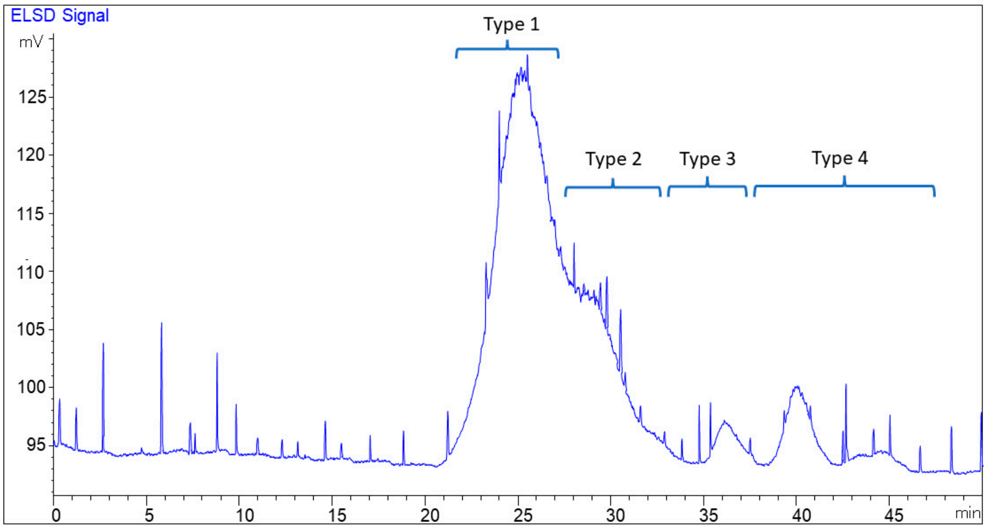

Figure S1. HPLC-ELSD chromatogram of GP pectin extracted with Oxalic Acid. On the figure are indicated the different types of fragments observed.

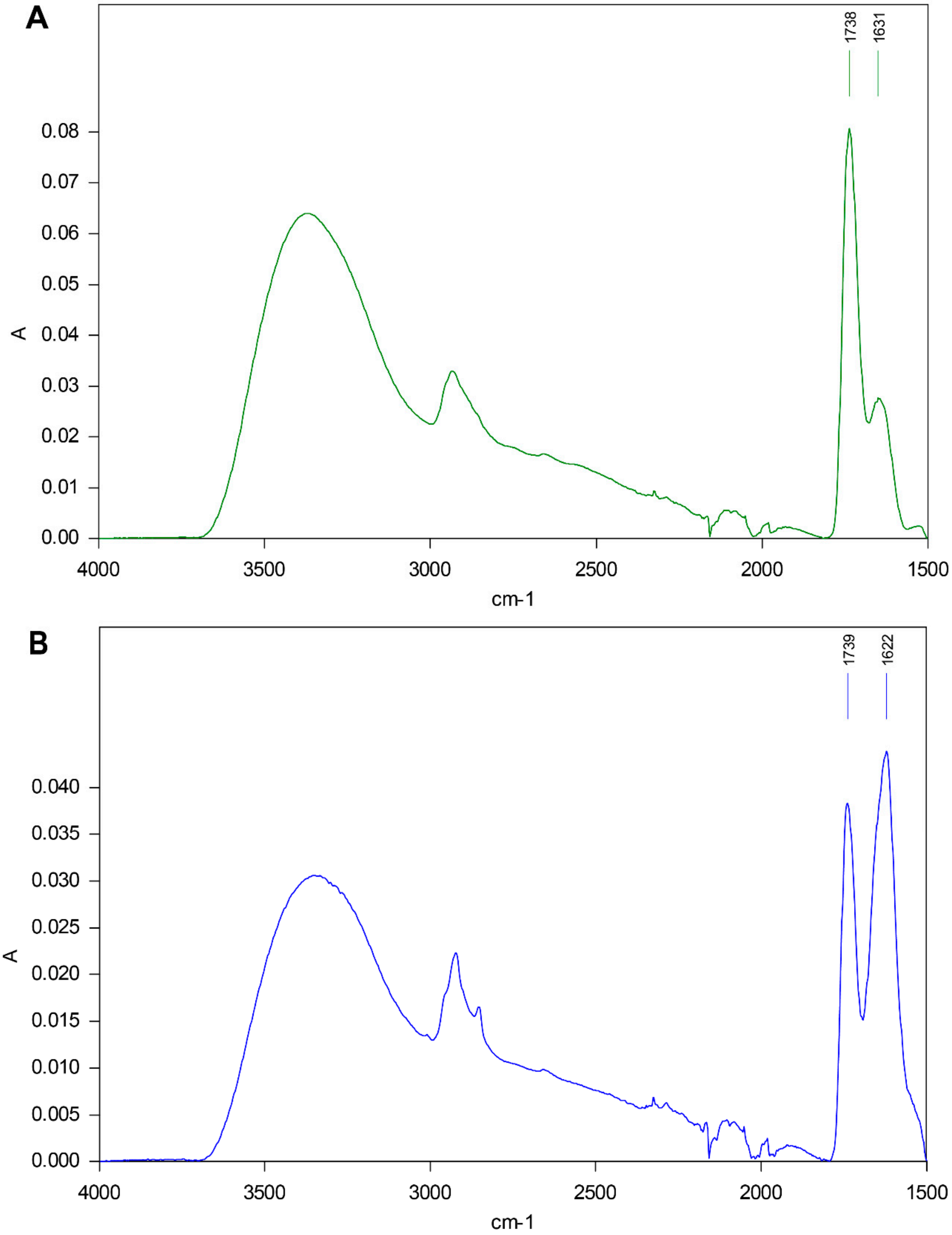

Figure S2. ATR-FTIR spectra (signal between 4000-1500 cm^{-1}) of pectin obtained with A) Nitric Acid, B) Oxalic Acid

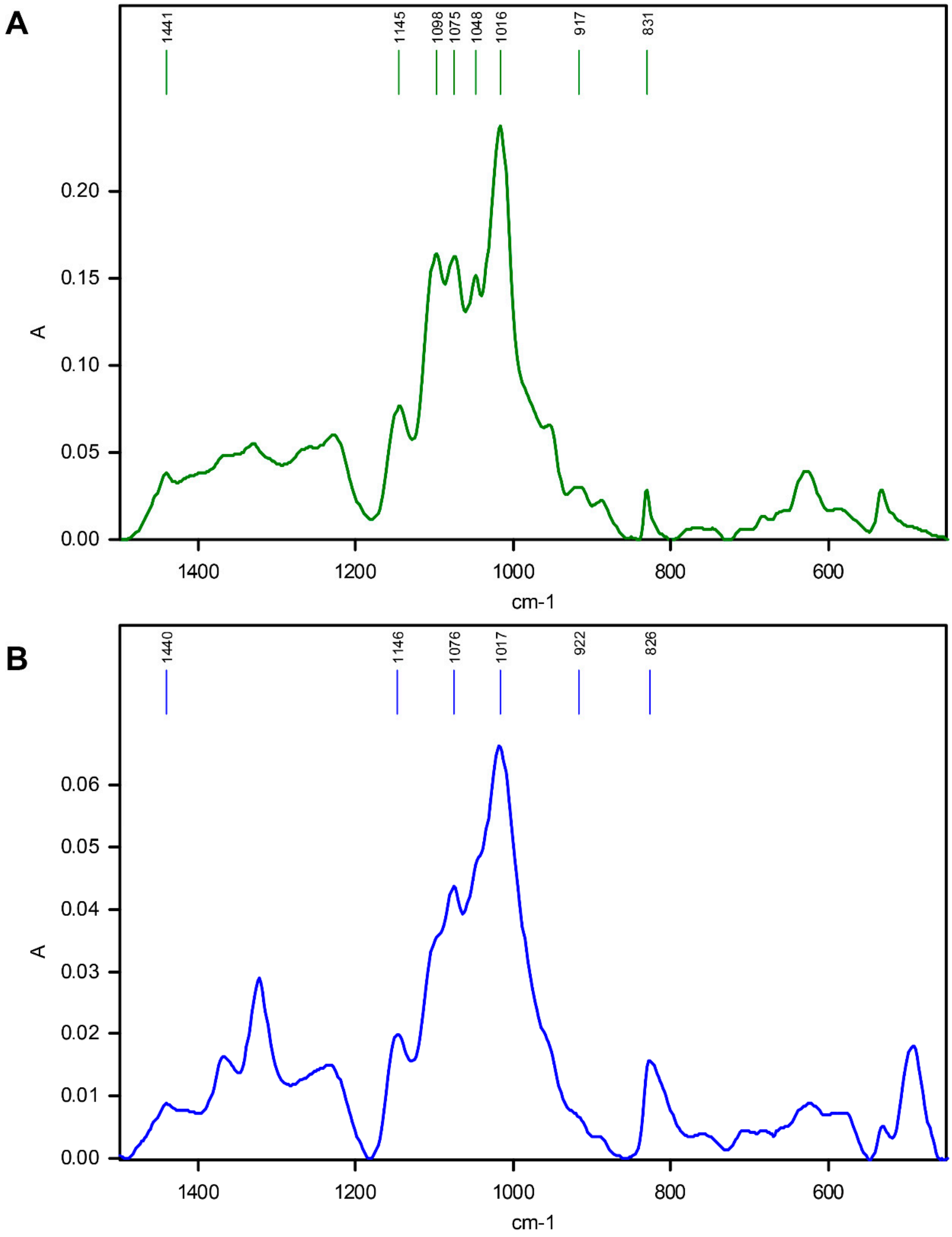

Figure S3. ATR-FTIR spectra (signal between 1500-450 cm^{-1}) of pectin obtained with A) Nitric Acid, B) Oxalic Acid
